# Supplementary material for: Incidence and Impact of Acute Kidney Injury after Liver Transplantation: A Meta-Analysis
Source: J Clin Med. 2019 Mar 17;8(3):372. doi: 10.3390/jcm8030372 (PMC6463182; doi:10.3390/jcm8030372)

Online supplementary data 1

Search terms for systematic review.

Databases: Ovid MEDLINE (697 articles)

1. exp acute kidney injury/

2. acute kidney injury.mp

3. AKI.mp

4. 1 or 2 or 3

5. liver.mp

6. hepatitc.mp

7. 5 or 6

8. transplant.mp

9. transplantation.mp

10. 8 or 9

11. 7 and 10

12. 4 and 11

Databases: EMBASE: (1,809 articles)

('liver transplantation' OR 'liver graft') AND 'acute kidney failure'

Database: Cochrane Databases (19 articles)

Search all text "acute kidney injury and liver transplantation"

Supplementary Figure S1: Funnel plot evaluating for publication bias evaluating incidence of post-LTx AKI.

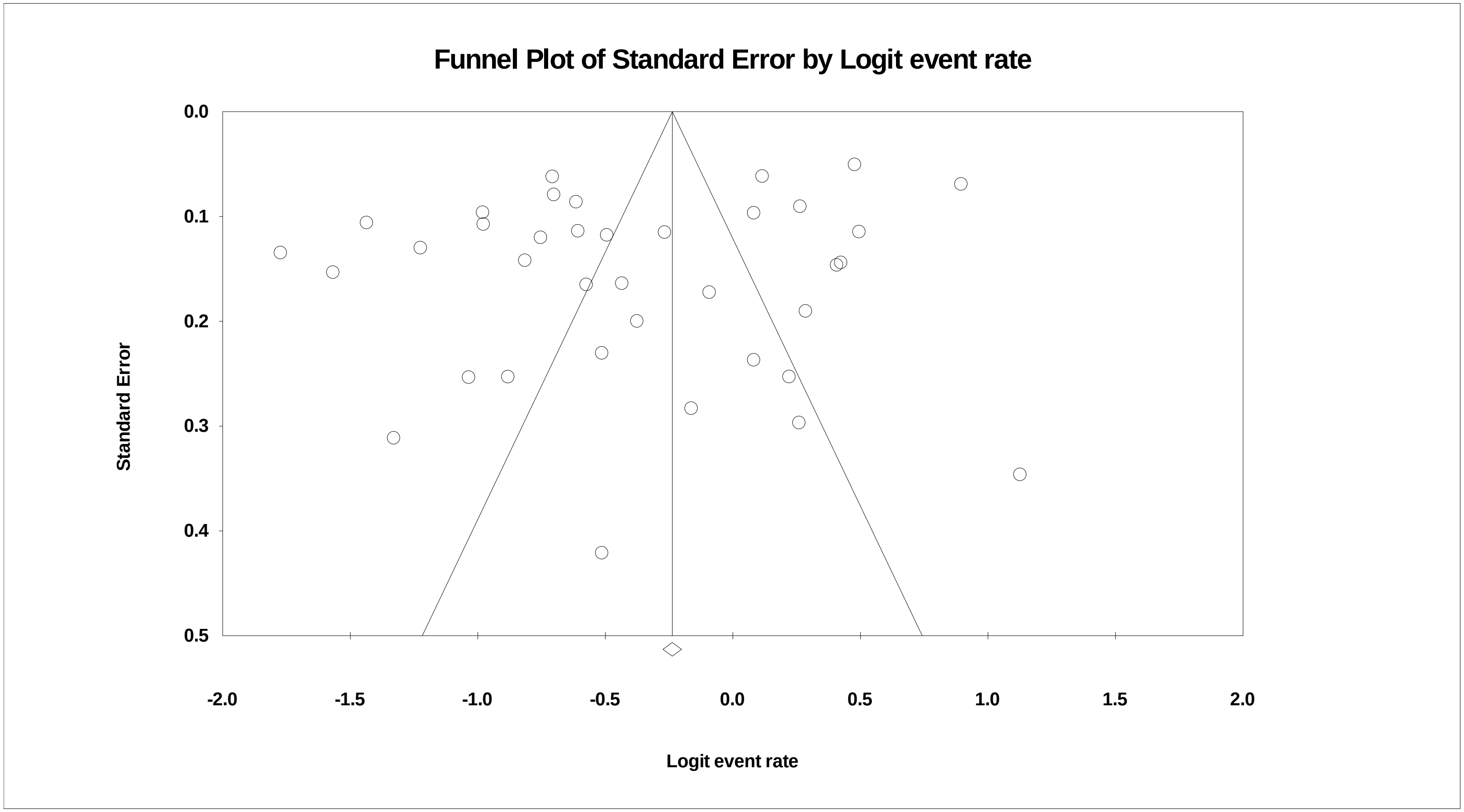

Supplement: Supplementary file 1 [file jcm-08-00372-s001.pdf]
